# Expression of Glypican 3 Is an Independent Prognostic Biomarker in Primary Gastro-Esophageal Adenocarcinoma and Corresponding Serum Exosomes

**DOI:** 10.3390/jcm8050696

**Published:** 2019-05-16

**Authors:** Mohammad Rahbari, Mathieu Pecqueux, Daniela Aust, Holger Stephan, Oliver Tiebel, Antonios Chatzigeorgiou, Torsten Tonn, Franziska Baenke, Venkatesh Rao, Nicole Ziegler, Helena Greif, Kuailu Lin, Juergen Weitz, Nuh Nabi Rahbari, Christoph Kahlert

**Affiliations:** 1 Department of Visceral, Thoracic and Vascular Surgery, University Hospital Carl Gustav Carus, Technical University Dresden, D-01307 Dresden, Germany; Mohammad.Rahbari@uniklinikum-dresden.de (M.R.); Mathieu.Pecqueux@uniklinikum-dresden.de (M.P.); Franziska.Baenke@uniklinikum-dresden.de (F.B.); Rao.Venkatesh@uniklinikum-dresden.de (V.R.); Nicole.Ziegler@uniklinikum-dresden.de (N.Z.); Helena.Greif@uniklinikum-dresden.de (H.G.); Kuailu.Lin@uniklinikum-dresden.de (K.L.); Juergen.Weitz@uniklinikum-dresden.de (J.W.); 2 Institute for Pathology, University Hospital Carl Gustav Carus, Technical University Dresden, D-01307 Dresden, Germany; Daniela.Aust@uniklinikum-dresden.de; 3 Tumor and Normal Tissue Bank of the University Cancer Center, Technical University Dresden, D-01307 Dresden, Germany; 4 Helmholtz-Zentrum Dresden-Rossendorf, Institute of Radiopharmaceutical Cancer Research, Bautzner Landstrasse 400, D-01328 Dresden, Germany; h.stephan@hzdr.de; 5 Institute of Clinical Chemistry and Laboratory Medicine, University Hospital Carl Gustav Carus, Technical University Dresden, D-01307 Dresden, Germany; oliver.tiebel@uniklinikum-dresden.de (O.T.); antonios.chatzigeorgiou@uniklinikum-dresden.de (A.C.); 6 Institute for Transfusion Medicine, German Red Cross Blood Donation Service North-East, D-01307 Dresden, Germany; t.tonn@blutspende.de; 7 Medical Faculty, University Hospital Carl Gustav Carus, Technical University Dresden, D-01307 Dresden, Germany; 8 German Cancer Consortium (DKTK) German Cancer Research Centre (DKFZ), D-69120 Heidelberg, Germany; 9 Department of Surgery, University Medicine Mannheim, Medical Faculty Mannheim, University of Heidelberg, Mannheim, D-68167 Mannheim, Germany; Nuh.Rahbari@umm.de

**Keywords:** exosomes, gastro-esophageal adenocarcinoma, glypican 3, prognostic biomarker, diagnostic biomarker

## Abstract

Exosomes are nano-sized membranous vesicles of endosomal origin that carry nucleic acids, lipids and proteins. The cargo of exosomes is cell origin specific and the release of these exosomes and uptake by an acceptor cell is seen as a vital element of cell-cell communication. Here, we sought to investigate the diagnostic and prognostic value of the expression of glypican 3 (*GPC3*) on primary gastro-esophageal adenocarcinoma (GEA) tissue (*tGPC3*) and corresponding serum exosomes (*eGPC3*). Circulating exosomes were extracted from serum samples of 49 patients with GEA and 56 controls. Extracted exosomes were subjected to flow cytometry for the expression of *eGPC3* and *GPC3* expression on primary GEA tissue samples was determined by immunohistochemistry and correlated to clinicopathological parameters. We found decreased *eGPC3* levels in GEA patients compared to healthy controls (*p* < 0.0001) and high *tGPC3* expression. This was significantly associated with poor overall survival (high vs. low *eGPC3*: 87.40 vs. 60.93 months, *p* = 0.041, high vs. low *tGPC3*: 58.03 vs. 84.70 months, *p* = 0.044). Cox regressional analysis confirmed *tGPC3* as an independent prognostic biomarker for GEA (*p* = 0.02) and *tGPC3* expression was validated in two independent cohorts. Our findings demonstrate that *eGPC3* and *tGPC3* can be used as potential diagnostic and prognostic biomarkers for GEA.

## 1. Introduction

Gastro-esophageal adenocarcinomas (GEA) are one of the most common causes of cancer-related mortality [1], despite an overall decline in incidence [2]. True non-cardia carcinomas and cancers of the esophagogastric junction develop asymptomatically and at time of diagnosis, most patients present with loco-regional or metastatic disease, limiting the possibility for curative interventions [3]. GEA is a heterogeneous disease with distinct clinical subtypes and the complex molecular mechanisms underlying the initiation and progression of these cancers is not very well understood. Thus, there is an urgent need to better characterize the molecular mechanism underlying this disease and develop effective screening systems to facilitate earlier diagnosis and treatment [3].

Liquid biopsies are a non-invasive tool to provide an easily accessible surrogate for tumor tissue and represent a promising approach to detect malignancies at early stage and monitor the response to treatment [4,5]. In gastric cancer patients, a large number of novel blood-based biomarkers have been reported for their potential role in clinical practice [6]. However, none of these have been established in the clinic for early detection or disease monitoring. The most commonly used biomarkers for GEA are serum carbohydrate antigen 19-9 (CA-19-9), carbohydrate antigen 72-4 (CA-72-4) and carcinoembryonic antigen (CEA), although it has been shown that they vary in their sensitivity and specificity and strongly depend on tumor burden [7].

Exosomes comprise a subgroup of extracellular vesicles, defined by their endosomal origin and size ranging from 20–150 nm [8]. As exosomes are spherical, lipid-bilayered vesicles, generated through a complex regulated process within multivesicular bodies, they may resemble the molecular landscape of the donor tissue and its pathologic characteristics [9]. Several studies have highlighted the crucial role that exosomes play in the pathobiology of GEA [10,11,12]. These vesicles carry nucleic acids, lipids and proteins which can act as signal molecules for intercellular communication [13], thereby promoting the activation of the PI3K-AKT and MAPK pathway, which significantly impacts cancer cell proliferation and invasion [10]. These characteristics of exosomes may provide unique opportunities for the detection of malignancies and identification of diagnostic and prognostic biomarkers [14].

Glypicans belong to a group of heparan-sulfate proteoglycans, a large family of plasma membrane associated glycoproteins, which are involved in the crosstalk between cancer cells and their microenvironment [15]. Exosomes express glypicans on their cellular surface [16] and recently glypican 1^+^ (*GPC1*) exosomes have been identified for the detection of pancreatic cancer independent of staging [17] in multiple studies [18,19], emphasizing the great potential of exosome-based biomarkers for gastrointestinal cancers. Glypican 3 (GPC3), another member of the glypican protein family, has also been shown to act as a regulator of different cellular functions including proliferation and differentiation [20]. However, there is conflicting data in the literature regarding the role of GPC3 in gastric and esophageal cancer. Several studies have reported, that GPC3 is an oncofetal protein, which is associated with the alpha-fetoprotein-producing (AFP) hepatoid phenotype of gastric adenocarcinoma and gastric adenocarcinoma of enteroblastic differentiation [21,22,23]. Intriguingly, the common gastric adenocarcinomas express the oncofetal proteins GPC3 and AFP [24]. In addition, 25% of esophageal adenocarcinomas (2/8) and 26.5% of esophageal squamous cell carcinoma (SCC; (5/19) showed increased expression of GPC3 [25] in contrast to previous studies that reported lower frequencies of positive staining for GPC3 in esophageal SCC. Moreover, Zhu et al. have reported that expression of glypican 3 is markedly decreased in gastric cancer but not in esophageal cancer [26]. This data supports the finding by Han et al. that GPC3 is a potential suppressor of metastasis as loss of GPC3 was associated with increased lymph node metastasis and poor overall survival (OS) [27].

Here in this study, we sought to examine the expression of GPC3 in primary GEA tissue and matched blood for the presence of GPC3 positive exosomes (*eGPC3^+^*). Given that the expression of glypicans is altered during tumorigenesis and glycoproteins have been suggested as a promising target of biomarkers in cancer [28], we want to determine whether GPC3 may serve as a non-invasive diagnostic and prognostic tool for GEA.

## 2. Materials and Methods

The study was in accordance with the reporting recommendations for tumor marker prognostic studies (REMARK) guidelines [29].

### 2.1. Patient Characteristics and Data Collection

The Ethics committee of the Technical University of Dresden approved the study (EK 96032017). The experimental cohort consisted of 49 patients with GEA who underwent surgical resection between 2007 and 2013 at the Department of General, Thoracic and Vascular Surgery, University Hospital Carl Gustav Carus, Technical University Dresden, Dresden, Germany. Blood serum samples collected from patients without evidence of neoplastic disease (n = 25) and healthy donors (n = 31) were included as the control cohort. Two independent patient cohorts were used as validation cohorts as previously described [30,31]. The publicly available database Human Protein Atlas [32] includes a human pathology atlas section with a metadata study of the TCGA transcriptome data of 354 patients. GPC3 was one of the 171 genes associated with an unfavorable diagnosis [30]. The expression cutoff was 3.16 FPKM. The second database analyzed for GPC3 mRNA levels of primary GEA tumors was the Kaplan Meier Plotter (n = 876 [31]). To generate the Kaplan Meier graphs, we used the default settings for affyID 209200, which is GPC3.

### 2.2. Blood Collection and Isolation of Serum Exosomes

Venous blood samples were taken immediately before surgical resection using serum separator tubes. The tubes were centrifuged at 2500 × g for 10 min and the serum was aliquoted and stored at −80 °C. Serum exosomes were isolated using the Total Exosome Isolation Reagent (Invitrogen^TM^, Thermo Fisher Scientific^®^, Waltham, MA, USA, Cat-Nr.: 4478360) according to the manufacturer’s instructions. Briefly, 40 µl isolation reagent was added to 200 µl of serum and vortexed. After incubation at 4 °C for 30 min, the samples were centrifuged at 10,000 × g for 10 min at room temperature (RT). The exosome pellet was retrieved after removal of the supernatant and resuspended in 100 µl 1x phosphate buffered saline (PBS). The presence of exosomes has been confirmed and validated with standard and fluorescent nanoparticle tracking analysis (NTA) (ZetaView^®^, Particle Metrix GmbH, Meerbusch, Germany), Western blotting with exosomal markers and transmission electron microscopy (TEM) (see sections below).

### 2.3. Nanoparticle Tracking Analysis (NTA) and Fluorescent Nanoparticle Tracking Analysis (fNTA)

Nanoparticle tracking analysis (NTA) was conducted using the ZetaView^®^ to determine the concentration and size of the extracted exosomes according to the manufacturer’s instructions. Additionally, the fluorescent mode of the ZetaView^®^ was employed to measure exosomal markers (CD9, CD63 and TSG101) of the extracted particles. For this analysis, 1 × 10^10^ exosomes in 100 µl 1x PBS were bound to 0.3 µm polystyrene latex beads (Sigma-Aldrich^®^, Merck, Darmstadt, Germany) and incubated for 15 min. Subsequently, this suspension was diluted to a total of 1 ml with 1x PBS and incubated on a horizontal shaker at 600 rpm for 60 min. The reaction was stopped by adding 100 mM glycine and 1% BSA in 1x PBS followed by centrifugation at 12,000 rpm for 3 min at RT. The exosome bound bead pellet was diluted in 40 µl 1x PBS. The final suspension was divided in two equal volumes. One aliquot was used for the detection of exosomal markers (CD9 monoclonal rabbit, dilution 1:20, Abcam, Cambridge, UK, Cat. Nr.: ab92726; CD63 polyclonal rabbit, dilution 1:20, Santa Cruz, Dallas, TX, USA, Cat. Nr. sc-15363; and TSG101, monoclonal mouse, dilution: 1:100, Abcam, Cat. Nr.: ab83). The second aliquot was used as a control for unspecific binding (secondary antibody alone). After 1 h of incubation, the reaction was stopped by adding a blocking solution of 100 mM glycine and 1% BSA in 1x PBS. Subsequently, the suspension was centrifuged at 12,000 rpm for 3 min at RT and the exosomes-bound beads were recovered as pellet. Alexa-488 secondary antibody (Life Technologies, Carlsbad, CA, USA; anti-rabbit: A11034 or anti-mouse: A11029) was added and incubated for 60 min at RT. After three washing steps in 1 x PBS/2% BSA, the suspension was diluted in 1 ml 1x PBS. The fluorescent exosome-bound beads (positive and negative control) were analyzed using the ZetaView^®^ (Particle Metrix GmbH, Meerbusch, Germany) with the ZetaView^®^ 8.03.04.01 Software.

### 2.4. Western Blot (Immunoblotting)

Immunoblot analyses were performed as previously described with minor modifications [33]. Briefly, exosomal total protein was harvested using RIPA buffer including 1x Halt™ Protease- and Phosphatase-Inhibitor-Cocktail (ThermoFisher Scientific, Waltham, MA, USA). Using Bradford quantification, 25 µg of protein was denaturized and separated by a 4–12% gradient Bis-Tris polyacrylamide gel electrophoresis (Invitrogen^TM^, ThermoFisher Scientific). The proteins were then transferred onto a nitrocellulose blotting membrane (Amersham^TM^ Protran^TM^, Amershan, UK) using a wet blotting approach. After blocking for 1 hour at RT, the blots were incubated with following primary antibodies: anti-CD9 (ERP2949) (Abcam), anti-CD63 (H-193) (Santa Cruz), anti-CD81 (NBP2-20564) (Novus Biologicals, Centennial, CO, USA), anti-TSG101 (4A10) (Abcam), anti-GPC-3 (AF2119, R&D Systems) and anti-calreticulin (Cell Signaling, Danvers, MA, USA; Cat. Nr. 2891) overnight at 4 °C on an orbital shaker. The next day, the blots were washed with 1 x PBS/0.05% Tween for at least 30 min, followed by the incubation with horseradish peroxidase (HRP) coupled secondary antibodies (Cell Signaling, anti-mouse Cat. Nr. 7076, anti-rabbit Cat. Nr. 7074) for 1 hour at RT. The blots were washed again and developed with Immobilon Western chemiluminescent HRP substrate using a G:Box XT4 imager (Syngene, Cambridge, UK).

### 2.5. Transmission Electron Microscopy (TEM)

Fixed specimens at an optimal concentration were placed onto 400-mesh carbon/formvar coated grids and allowed to absorb to the formvar for a minimum of 1 min. After rinsing in 1x PBS and distilled water the grids were allowed to dry and stained for contrast using uranyl acetate. The samples were viewed with a Tecnai Bio Twin transmission electron microscope (TEI) and images were taken with an AMT CCD Camera (Advanced Microscopy Techniques, Woburn, MA, USA).

### 2.6. Flow Cytometry Analysis (FC)

Exosomes were attached to 4 mm aldehyde/sulphate latex beads (Invitrogen^TM^) by mixing 100 µl of exosomes solution with 10 µl volume of beads for 30 min at 4 °C. This suspension was diluted to a total of 1 ml with PBS and incubated for 30 min rotating at RT. The reaction was stopped adding 100 mM glycine and 2% BSA in PBS and further rotating for 30 min at RT. Exosome-bound beads were washed once in 5% Milk in PBS and centrifuged for 1 min at 14,800× *g*, blocked with 10% BSA / 1x PBS by rotating for 30 min at RT, washed a second time in 2% BSA / 1x PBS and centrifuged for 1 min at 14,800× *g*, and incubated with anti-GPC3 (R&D Systems, Minneapolis, MN, USA; AF2119, 15 µl of antibody in 15 µl PBS) for 30 min at 4 °C. Beads were centrifuged for 1 min at 14,800× *g*, the supernatant was discarded and beads were washed in 2% BSA and centrifuged for 1 min at 14,800× *g*. Alexa-488 secondary antibodies (Life Technologies, 3 µl of antibody in 20 ml of 2% BSA) were incubated for 30 min at 4 °C. Incubation of the secondary antibody alone was used as control to identify the population of beads with GPC3-bound exosomes. The percentage of positive beads was calculated to the total number of beads analyzed per sample (20,000 events) and therein referred to as the percentage of beads with GPC3^+^ exosomes.

### 2.7. Immunohistochemistry (IHC)

Immunohistochemical staining of GPC3 on formalin-fixed, paraffin-embedded whole tissue sections was performed as described previously [34]. All slides were stained in the Autostainer Lab Vision 408S using the anti-GPC3 antibody (dilution 1:25 in 1x PBS, MSK067-05; Zytomed, Berlin, Germany). The endogenous peroxidase was blocked with peroxidase block (CellMarque, Merck) and BrightVision + Poly-AP-anti mouse/rabbit IgG biotin-free antibody kit (VWRKDPVB110AP; ImmunoLogic, Palo Alto, CA, USA) was used. Two independent researchers blindly scored the expression of GPC3 and consensus was reached for each slide. The staining intensity of GPC3 was classified as absent: 0, weak: 1, medium: 2 and strong: 3. The scoring for GPC3 expression was based on several publications assessing GPC levels in a broad spectrum of tumor types [35,36,37,38].

### 2.8. CA19-9, CA72-4 and CEA and Immunoassays

Protein levels were measured in patients with GEA, individuals with non-malignant disease and healthy donors using a Human Sandwich-Chemiluminescence-Immunoessay for the cancer antigen CA19-9 (Diasorin, Saluggia, Italy; 314171), CA72-4 (Cobas, Roche, Basel, Switzerland; ms_11776258122V12.0) and the CEA (Diasorin, 314311) according to the manufacturer’s directions.

### 2.9. Statistical Analyses

All statistical analyses have been carried out with the IBM SPSS Statistics software version 25 (SPSS, Chicago, IL, USA), GraphPad Prism version 6 (GraphPad Software, San Diego, CA, USA) and MedCalc^®^ (MedCalc Software bvba, Ostend, Belgium, Version 18.11.6). Categorical data were expressed as absolute and relative frequencies and compared using Chi-square-test or Fisher´s exact test. Continuous data were reported as arithmetic mean with the corresponding standard deviation. Analysis of variance (ANOVA) tests or student´s t tests were performed to calculate differences of multiple serum factors in human serum samples as wells as the area under the curve (AUC) through receiver operating characteristics (ROC) curve analysis. Differences between ROC curves were calculated MedCalc^®^ software. Overall survival was calculated from the date of surgery for GEA to the date of death or the date of last follow-up information. The Kaplan-Meier method was used to construct survival curves that were compared using the log-rank test. A Cox proportional hazards regression analysis was used to assess independent predictors of overall survival. A *p*-value ≤ 0.05 was considered statistically significant.

## 3. Results

### 3.1. Study Design and Study Population

To conduct the study, we used a cohort of 49 patients with GEA who underwent surgical resection at the Department of General, Thoracic and Vascular Surgery of the University Hospital Carl Gustav Carus Dresden, Technical University Dresden, Dresden, Germany. Clinicopathologic characteristics of the GEA patients are summarized in Table 1.

The pathological analysis revealed that the majority of patients were male (34/49), the intestinal, diffuse or mixed type of GEA was 47%, 29% and 10% of the cases according to the Lauren classification. Most of the patients presented with GEA of UICC stage I and II (37/49), tumor size of T1 and T2 (34/49) and a lymph node status of N0 and N1 (39/49). Six out of 49 patients had synchronous, metastatic disease and the median follow-up duration was 36.4 (0.1–93.3) months. 

### 3.2. Confirmation of the Presence of Exosomes

**Figure 1:**
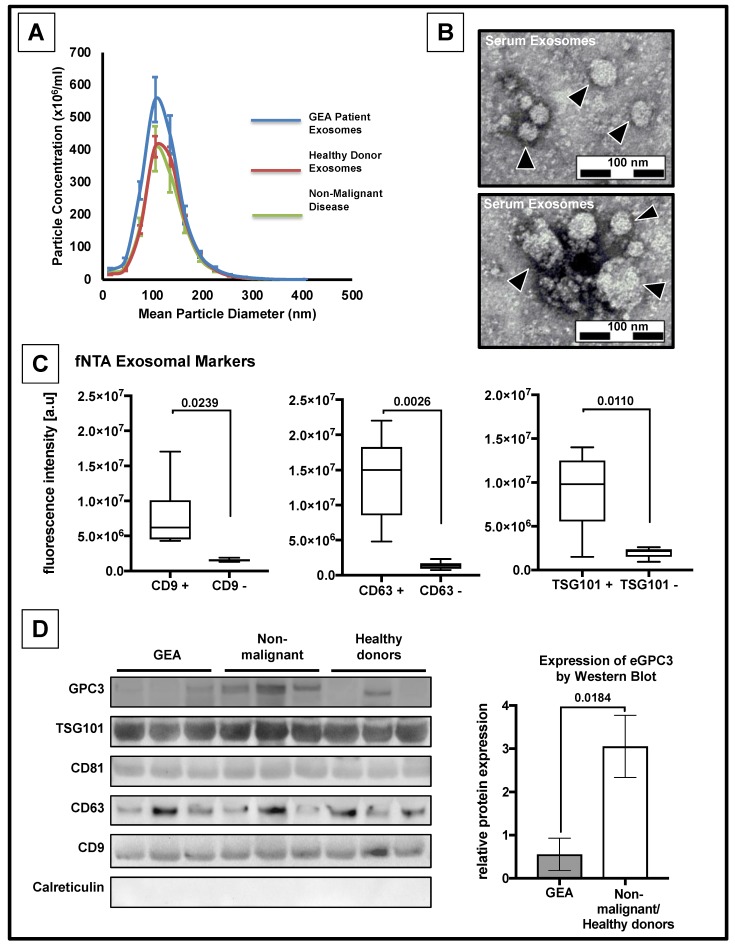
Decreased expression levels of *eGPC3* in patients with gastro-esophageal adenocarcinomas (GEA). (**A**) Nanoparticle tracking analysis (NTA) diagram of nanoparticle concentration and average particle size from serum samples of patients with GEA (n = 9; blue line), healthy donors (n = 9; red line) and patients with non-malignant disease (n = 9; green line). (**B**) Transmission electron microscopy (TEM) images of serum derived vesicles (black arrowheads). (**C**) Quantification of exosome-associated proteins CD9, CD63 and TSG101 determined by fluorescent NTA (*p* < 0.05, two-paired student´s t-test). (**D**) Protein expression analyses of exosome-derived GPC3, TSG101, CD81, CD63, and CD9. Calreticulin was used as a marker for cytoplasm-derived contamination (bottom panel). Representative quantification of GPC3 levels of serum-derived exosomes from patients with GEA compared to serum-derived exosomes from healthy donors and patients with non-malignant disease (right panel, *p* < 0.05 two-paired student´s t-test).

Initially, we sought to determine whether it is possible to measure exosomes from GEA patients. Therefore, serum and tissue samples from patients with GEA, serum samples from patients with non-malignant diseases (n = 25) and healthy donors (n = 31) were collected and subjected to exosome extraction. We performed nanoparticle tracking analysis (NTA) of selected specimens of 44 patients with GEA, 25 patients with non-malignant disease and 29 healthy donors. Moreover, we confirmed medium size of the isolated particles of about 105 nm in diameter in nine representative specimens of each subgroup of patients and healthy donors (Figure 1A), matching the size of exosomes ranging from 20 to 150 nm [8]. We observed an increased concentration of exosomes in patients with GEA in comparison to patients with non-malignant disease or healthy donors (*p* < 0.05, respectively) (Supplementary Figure S1A). In contrast, the median size of exosomes was significantly increased in healthy donors and patients with a non-malignant disease in comparison to patients with GEA (*p* < 0.05, respectively) (Supplementary Figure S1B). By using Transmission Electron Microscope (TEM), we were able to visualize the presence of vesicular structures with a bilayered lipid membrane and within the size range of exosomes (Figure 1B). To validate that these vesicles are exosomes, we analyzed the expression of exosomal marker proteins using immunoblotting and fluorescent nanoparticle tracking analysis (fNTA) (Figure 1C,D). We detected the presence of exosome-associated markers TSG101, CD9 and CD63 with both approaches, whilst CD81 was only detected by immunoblotting. To assess the expression of GPC3 on serum derived exosomes (*eGPC3*) in these samples, we performed immunoblot analysis of GPC3. *eGPC3* was enriched in the samples from healthy donors and patients with a non-malignant disease compared to the samples from patients with GEA (*p* < 0.05, Figure 1D). To eliminate cross-contamination of proteins of endosomal origin, we stained for calreticulin and found no detectable protein expression for calreticulin suggesting that the levels of *eGPC3* are indeed from the exosomes. Taken together, we were able to visualize exosomes in the serum of patients with GEA according to the recommendations for the characterization of exosomes [39,40].

### 3.3. eGPC3 Outperforms Current Serum Biomarkers of GEA And Negatively Correlates with Overall Survival

**Figure 2:**
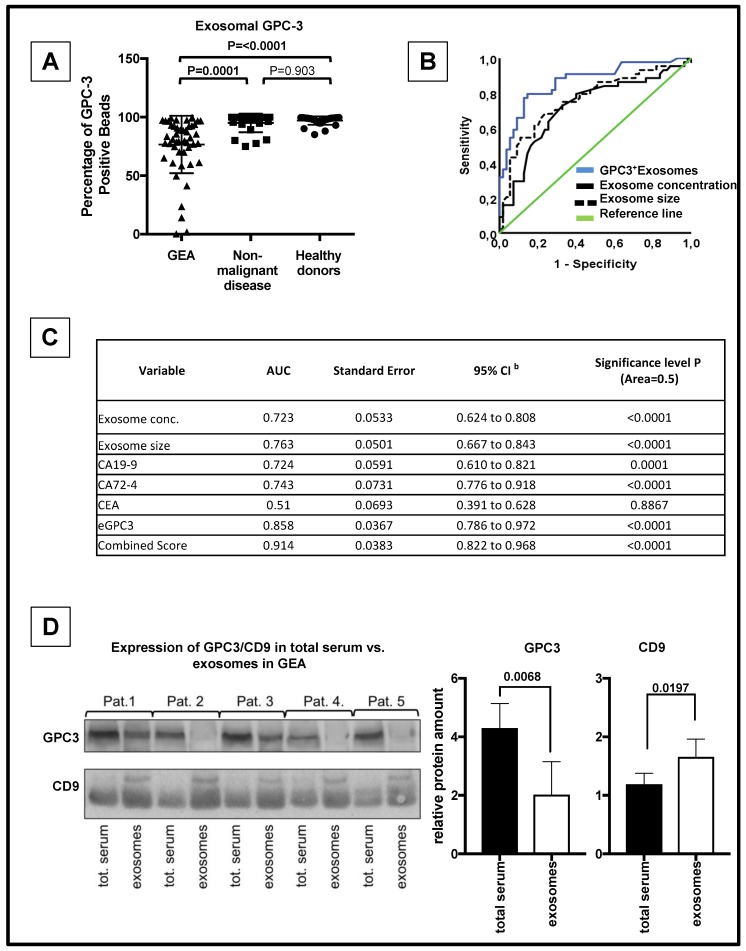
Decreased serum levels of *eGPC3* in GEA patients. (**A**) Dot plot analysis of the percentage of GPC3 positive exosome-bound latex beads in serum from patients with GEA, patients with non-malignant disease and healthy donors. (**B**) Receiver operating characteristics (ROC) curve analysis of *eGPC3* (blue), exosome concentration (black), and exosome size (dashed line) for patients with GEA vs. patients with non-malignant disease and healthy donors. (**C**) ROC curve analysis showing the area under the curve (AUC) for *eGPC3*, exosome concentration, exosome size, serum CA 19-9, serum CA 72-4, serum CEA and a combined score (eGPC3* CA 72-4)/CA 19-9) for patients with GEA vs. patients with non-malignant disease and healthy donors. (**D**) Protein expression analysis of GPC3 and CD9 in total serum and corresponding exosomes from five patients with GEA (left panel). Quantification of GPC3 and CD9 protein expression is shown on the left panel (right panel; *p* < 0.05).

To determine whether *eGPC3* expression can be used as a diagnostic marker of GEA, we conducted flow cytometry analysis of GPC3 on exosome-bound latex beads in healthy donors (n =3 1), patients with a non-malignant disease (n = 25) and patients with GEA from our Dresden cohort (n = 49). The count of GPC3 positive exosome-bound latex beads was significantly lower in GEA patients compared to healthy donors or patients with non-malignant disease (*eGPC3*: 97.04% in healthy donors, 95.03% in patients with non-malignant disease, 76.56% in GEA patients, ANOVA, *p* < 0.0001) (Figure 2A, Supplementary Figure S1C) confirming the results of the immunoblot analysis of exosome GPC3 shown in Figure 1D. ROC curve analysis was performed and resulted in an AUC of 0.85 with a sensitivity of 85.7% and a specificity of 75.5% for patients with GEA vs. control (healthy donors and patients with a non-malignant disease) (Figure 2B,C). In contrast, protein expression of routine biomarkers including CEA, CA 72-4 and CA 19-9 did not show any significant difference between our three different sample cohorts (Supplementary Figure S2A–C). A pairwise comparison of ROC curves revealed that the AUC of CEA and CA 19-9 was significantly inferior to the AUC of *eGPC3* (*p* < 0.05, respectively) (Figure 2C, Supplementary Figure S2D,E, and Supplementary Figure S3A). In contrast, pairwise comparison of ROC curves between *eGPC3* and CA 72-4 failed to be significant (*p* = 0.09) (Figure 2C, Supplementary Figure S2F and Supplementary Figure S3A). Intriguingly, by conducting a ROC curve analysis using a combined score of *eGPC3*, CA 72-4 and CA 19-9 ((eGPC3* CA 72-4)/CA 19-9), our data analysis yielded an AUC of 0.914 with a sensitivity of 80.6% and a specificity of 97.0% (Figure 2C). This combined score outperformed significantly the AUC of CA 72-4 and CA 19-9 (*p* < 0.05) but was not significantly increased when compared to the AUC of *eGPC3* (*p* = 0.84) (Supplementary Figure S3A).

To further elucidate the decreased expression of *eGPC3* in patients with gastric cancer, we sought to compare the expression of GPC3 and CD9 in total serum and corresponding isolated exosomes from five patients with GEA. Protein expression analysis showed increased GPC3 levels in the total serum fraction compared to the exosomal fraction (*p* < 0.05) (Figure 2D). In contrast, we found the exosomal marker CD9 was significantly elevated in the exosomal fraction than in the total serum fraction (*p* < 0.05) (Figure 2D). These data might suggest that *eGPC3* in patients with GEA can be cleaved by different proteases in the bloodstream and is therefore mainly present in a soluble form.

### 3.4. eGPC3 Negatively Correlates with tGPC3 and Is Associated with Dismal Overall Survival

We next sought to determine whether the tissue expression of GPC3 (*tGPC3*) in our GEA patient cohort demonstrated any correlation with the *eGPC3* levels. *tGPC3* expression was examined at the protein level in paraffin embedded healthy appearing gastric tissue adjacent to the tumor and primary GEA tumors by immunohistochemistry. Expression of GPC3 was found in 79.6% of all cases (39/49) and absent in 20.4% (10/49) compared to healthy tissue, which only showed positive staining in 8% of cases (Supplementary Figure S3B, representative figures, right upper panel). Next, we dichotomized the positive GPC3 expression according to the staining intensity (Low: staining intensity 0–1 vs. high: staining intensity 2–3) and found 28.6% of all cases with low staining intensity and 51% of cases with strong staining intensity (Supplementary Figure S3B, representative images). Spearman correlation analysis showed a strong inverse correlation between expression of tGPC3 and GPC3 on exosome-bound latex beads in patients with GEA (correlation coefficient: −0.304, *p* = 0.034).

GPC3 has been described as a biomarker in several other cancer types. Therefore, we sought to identify clinical and histopathological prognostic markers in our cohort including the expression of exosomal and tissue GPC3. We correlated the expression of *tGPC3* and *eGPC3* to the clinicopathological data that was available. A summary of the results is shown in Table 1. We observed a significant correlation between tumor grade and high expression of *tGPC3* (*p* = 0.050) whereas none of the other clinical or histopathological parameters showed a significant association. We further analyzed if grade 1 and 2 tumors showed a differential expression of *tGPC3* and *eGPC3* versus grade 3 and 4 tumors. However, this analysis revealed that there is no tumor grade-specific difference regarding *tGPC3* or *eGPC3* (Supplementary Figure S3C,D). Moreover, the Chi-Square test revealed that there is no significant correlation between tumor grading and tissue or exosome expression of GPC3 (Supplementary Figure S3E,F).

GPC3 has been reported to be a prognostic marker in several other cancer types. Therefore, we sought to identify clinical and histopathological prognostic marker in our cohort including the expression of exosomal and tissue GPC3. Univariate analysis revealed that lymph node status (*p* = 0.014), tumor size (*p* = 0.003), M stage (*p* = 0.003), L stage (*p* = 0.003), V stage (*p* = 0.001) and Resection status (*p* = 0.009) were associated significantly with overall survival (OS) (Table 2).

**Figure 3:**
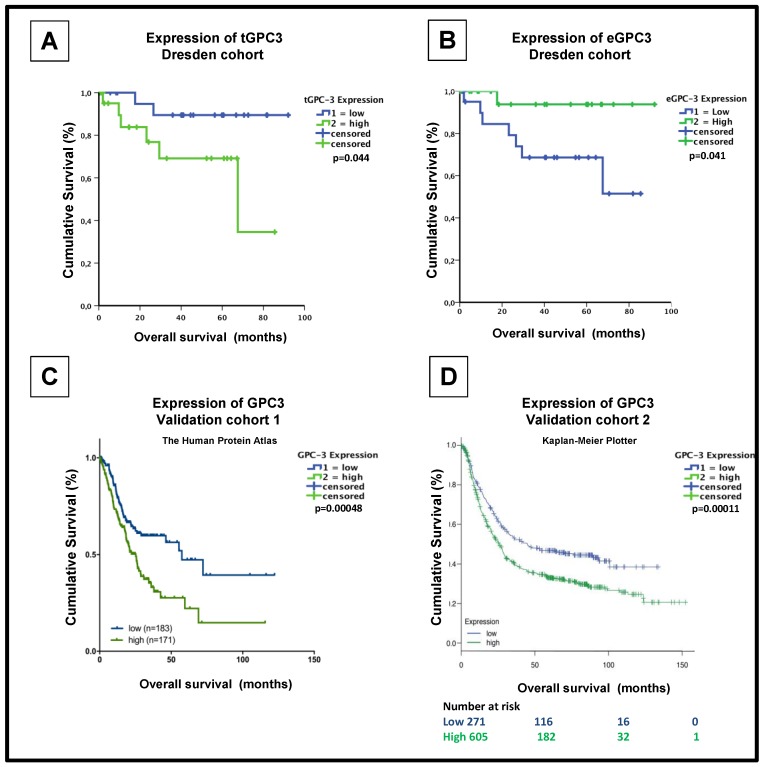
Expression of GPC3 in GEA tumor tissues correlates to poor overall survival. (**A**) Kaplan-Meier curve (months) from Dresden GEA cohort. High expression of GPC3 protein levels (green line) on GEA tissue conferring a poorer prognosis compared to patients with low GPC3 expression (blue line). (**B**) Kaplan-Meier curve (months) depicting overall survival in patients with high (green line) and low (blue line) percentage of expression of exosome-bound latex beads stained against GPC3. (**C**) Kaplan-Meier curve (months) from the Human Protein Atlas conferring overall survival in patients with high (green line) and low (blue line) expression of GPC3 in primary GEA cancer (n = 354; source: v18.proteinatlas.org/ENSG00000147257-GPC3/pathology) [30]. (**D**) Kaplan-Meier curve (months) depicting overall survival in patients with high (green line) and low (blue line) mRNA expression of GPC3 in gastric cancer (n = 876; source: [31])

When we compared the differential expression of *tGPC3*, we found that it was associated with a dismal prognosis in patients with GEA (low expression of *tGPC3*: median OS: 84.7 months, high expression of *tGPC3*: median OS: 58.0 months, *p* = 0.044.) (Figure 3A, Table 2). Interestingly, univariate analysis showed that high expression of GPC3 on exosome-bound latex beads was associated with improved OS (low expression of *eGPC3*: median OS: 60.926 months, high expression of *eGPC3*: median OS: 87.403 months, *p* = 0.041) (Figure 3B, Table 2) Further univariate analysis revealed that there was no significant correlation between aberrant expression of CEA, CA 72-4 or CA 19-9 and overall survival (*p* > 0.05, respectively, Supplementary Figure S4A–C).

In multivariate analysis, high expression of *tGPC3* remained formal statistical significance as a prognostic marker for poor OS in patients with GEA (*p* = 0.026; Table 3). However, multivariate analysis failed to reach the formal statistical significance between expression of GPC3 on exosome-bound latex beads and OS (*p* = 0.607; Table 3). To confirm the prognostic impact of *tGPC3* from our GEA cohort, we used data from two independent publicly available databases. A cohort of 354 patients with GEA from the Human Protein Atlas [32] showed increased expression of *tGPC3*, which correlated with a poor OS (low expression of tGPC3: 5-year survival: 47%, high expression of *tGPC3*: 5-year survival: 22%, *p* < 0.0001; Figure 3C) [30]. The second validation cohort of 876 patients with GEA [31] also revealed that patients with high GPC3 mRNA levels had significantly lower OS (low expression of *tGPC3*: median overall survival: 45.1 months, high expression of *tGPC3*: median overall survival: 26 months, *p* = 0.00011; Figure 3D) [31].

## 4. Discussion

In this study, we evaluated the expression of GPC3 on serum-derived exosomes and corresponding primary tumor tissue in patients with GEA. We demonstrate that increased expression of GPC3 in GEA tissue is associated with decreased overall survival in the Dresden cohort and two independent cohorts including a total of 1279 patients. 

These data contradict two previously published reports that have proposed GPC3 acts as a tumor suppressor gene. The expression of GPC3 was markedly decreased in gastric but not in esophageal cancer [26], suggesting an active downregulation of GPC3 in gastric tumor cells. However, Zhu et al. only examined the RNA expression of GPC3 with no further investigation of whether mRNA is functionally expressed on the protein level [26]. The influence of post-translational modification may explain the discrepancy between our results and the findings by Zhu et al. In addition, Zhu et al. have investigated the expression of GPC3 in esophageal squamous and adenocarcinomas, whilst our cohort only consisted of gastro-esophageal adenocarcinomas. The difference in these histological subtypes may explain the discrepancy between Zhu et al. and our results. Additionally, Han et al. reported that GPC3 is absent in invasive gastric tumors and lymph node metastases implying GPC3 is a potent metastasis suppressor in GEA [27]. Their cohort included a heterogeneous group of patients with gastric adenocarcinoma including patients with signet ring cell carcinoma. The expression of GPC3 was much lower in signet ring cell carcinomas in comparison to other adenocarcinomas. As this histological subtype is known to be more aggressive and associated with a poor prognosis [41], it may have biased the results and led to a different conclusion with regards to our study. A recent study by Yamazawa and colleagues has investigated a panel of stem cell and oncofetal markers including GPC3 in 386 patients with gastric cancer [42]. In concordance with our current data, the authors show that positive staining for these panel markers including GPC3 was associated with poor prognosis and was an independent risk factor for disease-free survival. Intriguingly, our evaluation between the expression of GPC3 and histopathological data revealed high expression of tissue-derived GPC3 correlated with a lower tumor grade. This data is in contradiction to previous studies that show a positive correlation between the expression of GPC3 and tumor grading in other solid malignancies such as hepatocellular or urothelial carcinomas [43,44]. Our opposing findings could be explained by intratumoral sampling variability as it has been shown for hepatocellular carcinomas [45]. The variability of tumor grading in a heterogenous tumor bulk can lead to a biased interpretation of the results. To further assess whether the expression of GPC3 can be employed as a differential diagnostic marker for tumor grading, additional studies would be necessary with a more exclusive focus on the tissue expression of GPC3 in gastric cancer.

Our study shows that GPC3 is significantly decreased on serum-derived exosomes from GEA patients in comparison to healthy donors or patients with a non-malignant disease, when we analyzed the occurrence of GPC3 on circulating extracellular vesicles. These data concur with previous studies that provide evidence for a discrepant expression of proteins between tumor cells and corresponding serum exosomes. For example, CD47 is highly expressed in breast cancer tissue samples [46]. However, when Kibria et al. measured the expression of CD47 on circulating exosomes, this surface marker was unexpectedly detected in healthy donors and only minimal CD47 expression was observed in circulating exosomes from breast cancer patients [47]. Likewise, while the epithelial cell adhesion molecule (EpCAM) is strongly expressed in breast cancer tissue [48], it seems to be decreased on serum-derived exosomes of breast cancer patients [49]. Thus far, in our study, we have not further elucidated the inverse expression of GPC3 in tumor tissue and corresponding serum exosomes in GEA. However, we show that GPC3 is more abundant in total serum than in the exosomal fraction. Based on these findings, it could be hypothesized that GPC3 can be cleaved by different proteases in the bloodstream. This mechanism has been observed by Rupp et al. who have provided data that the expression of EpCAM on serum-derived exosomes is decreased since it is cleaved from exosomes via metalloproteinases in the circulation [49]. In this context, our findings would not be contradictory to studies that have described an overexpression of GPC3 in the serum of patients with hepatocellular carcinoma [50,51], assuming that the soluble form of GPC3 was measured. An alternative theory might be that the significant inverse correlation of *tGPC3* and *eGPC3* in patients with GEA is based on the active suppression of the presence of GPC3 in the systemic circulation by tumor cells. This suppression would prevent the degradation of these exosomes in the blood in order to deliver their tumorigenic load to distant organs for the formation of pre-metastatic niches. Moreover, the presence of GPC3 on serum exosomes in patients with GEA could be suppressed through a modified loading process of protein cargo including GPC3 into multivesicular bodies and exosomes. Villarroya-Beltri et al. described the modification of the loading process of exosomes in cancer cells [52]. This alternate loading of cancer cell derived exosomes may in turn contribute to our observation of the accumulation of GPC3 in GEA cancer cells. In this context, the low exosomal GPC3 levels could be linked to a failed release resulting in an intracellular accumulation, as we have observed in our cohort and as it has been described in the independent validation cohorts.

However, despite the lack of more profound information about the functional role of GPC3-positive exosomes in GEA, we show a potential role as a minimal-invasive prognostic biomarker that can be utilized for molecular risk stratification and personalized postoperative therapy guidance. Moreover, we show that low expression of GPC3 on serum exosomes is superior to standard serum markers such as CEA or CA 19-9 in order to discriminate between patients with GEA and the control group. Additionally, we can show that a combined panel of serum biomarkers including exosomal GPC3 can increase the diagnostic power for the non-invasive discrimination of patient with GEA vs. healthy donors of patients with a non-malignant disease. Therefore, though in isolation, GPC3 may not have clinical utility, it might be an interesting candidate for a multi-parametric exosomal biomarker panel to facilitate the diagnosis of GEA. For clinical integration of *eGPC3* and *tGPC3* as biomarkers for GEA, however, further validation of the above given findings in prospective clinical trials is necessary.

## Figures and Tables

**Table 1:** Clinicopathologic characteristics of included patients.

Characteristics	Total Number of Cases	eGPC3			*tGPC3*		
		*eGPC3* ^low^	*eGPC3* ^high^	*P*-Value	*tGPC3* ^low^	*tGPC3* ^high^	*P*-Value
**Age**							
< median = 63 years	24	15	9	0.156	11	13	0.778
≥ median = 63 years	25	10	15		13	12	
**Gender**							
Male	34	16	18	0.538	18	16	0.538
Female	15	9	6		6	9	
**Classification of Lauren**							
Intestinal	23	11	12	0.342	9	14	0.560
Diffuse	14	8	6		8	6	
Mixed	5	1	4		2	3	
Missing	7	5	2		5	2	
**UICC stage**							
I and II	37	18	19	0.742	20	17	0.321
III and IV	12	7	5		4	8	
**Tumor size**							
T1 and T2	34	16	18	0.538	17	17	1.000
T3 and T4	15	9	6		7	8	
**Lymph node status**							
N0 and N1	39	9	15	0.089	20	19	0.572
N2 and N3	10	16	9		4	6	
**Metastasis status**							
M0	43	20	23	0.189	23	20	0.189
M1	6	5	1		1	5	
**Resection margin status**							
R0	43	20	23	0.189	22	21	0.667
R1 and R2	6	5	1		2	4	
**Tumor differentiation**							
G1 and G2	17	9	8	0.751	4	13	**0.050**
G3 and G4	22	10	12		13	9	
**CEA**							
< Median	21	9	12	0.538	12	9	0.215
> Median	20	11	9		7	13	
**CA 19-9**							
< Median	21	11	10	0.758	8	13	0.354
> Median	20	9	11		11	9	
**CA 72-4**							
< Median	24	12	12	0.732	11	13	0.292
> Median	12	5	7		3	9	
**Adjuvant therapy**							
Yes	22	13	8	0.244	11	16	0.244
No	27	11	16		13	8	
**Neoadjuvant therapyes**							
Yes	27	16	11	0.256	9	13	0.393
No	22	9	13		15	12	

Abbreviations: Glypican 3 (GPC3), tissue expression of GPC3 (*tGPC3*) and corresponding serum exosomes (*eGPC3*), carcinoembryonic antigen (CEA), carbohydrate antigen (CA), union of international cancer control (UICC). Significant p values are highlighted using bold font.

**Table 2:** Univariate analysis (log-rank test) of prognostic parameters in GEA for median overall survival.

Characteristics				95%-Confidence-Interval		
	No. of Cases	Mean Survival (Months)	Standard Error	Lower Limit	Upper Limit	*P*-Value
**Adjuvant Chemotherapy**						
Not received	27	62.6	4.3	54.2	71.0	0.386
Received	22	69.6	8.4	53.1	86.1	
**Neoadjuvant Chemotherapy**						
Not received	22	78.1	7.3	63.8	92.4	0.746
Received	27	68.8	6.5	56.1	81.4	
**Lymph node status**						
Negative	24	87.9	4.0	80.0	95.8	**0.014**
Positive	25	56.2	7.4	41.6	70.7	
**Tumor size**						
T1 and T2	34	83.1	4.8	73.7	92.4	**0.003**
T3 and T4	15	38.7	7.1	24.7	52.6	
**M stage**						
M0	43	76.2	5.3	65.8	86.6	**0.003**
M1	6	9.7	0.000	9.7	9.7	
**L stage**						
L0	27	87.2	4.4	78.6	95.7	**0.003**
L1	20	52.5	8.9	35.0	70.0	
**V stage**						
V0	39	81.6	4.8	72.3	91.0	**0.001**
V1	8	26.4	7.1	12.5	40.2	
**Tumor differentiation**						
G1 and G2	17	70.4	6.2	58.3	82.6	0.432
G3 and G4	22	71.1	8.0	55.4	86.8	
**UICC stage**						
I and II	37	76.4	5.7	65.2	87.5	0.392
III and IV	12	46.7	8.1	30.7	62.6	
**Resection status**						
R0	43	77.8	5.2	67.50	88.0	**0.009**
R1 and R2	6	24.0	8.6	7.1	40.9	
***eGPC3***						
Low	25	60.9	7.4	46.4	75.4	**0.041**
High	24	87.4	4.5	78.6	96.2	
***tGPC3***						
Low	24	84.7	4.9	75.0	94.4	**0.044**
High	25	58.0	8.2	41.9	74.2	

**Table 3:** COX proportional hazard models for multivariate analyses of *eGPC3* and *tGPC3* for overall survival.

Characteristics				95%-Confidence-Interval	
	Category	*P*-Value	Hazard Ratio	Lower	Upper
***eGPC3***	High vs. low	0.361	0.306	0.024	3.890
***tGPC3***	High vs. low	**0.026**	30.334	1.497	614.752
**N**	N0 vs. N+	0.781	0.635	0.026	15.587
**M**	M0 vs. M1	0.992	0.000	0.000	0.000
**L**	L0 vs. L1	**0.016**	68.320	2.204	2118.091
**V**	V0 vs. V1	0.473	0.462	0.056	3.816
**R**	R0 vs. R+	0.158	13.888	0.359	537.946

## Supplementary Materials

The supplemental data is available online at https://www.mdpi.com/2077-0383/8/5/696/s1, Figure S1: Comparison of mean exosome concentration and mean diameter in GEA patients, patients with non-malignant disease and healthy donors and representative histograms of flow cytometry analysis. Figure S2: Diagnostic significance of the expression of current laboratory markers for GEA in venous blood. Figure S3: Comparison of the AUC of ROC curves and impact of tumor grading on *eGPC3* and *tGPC3* expression. Figure S4: Expression of current laboratory markers for GEA in venous blood in association to overall survival outcomes.
