# Supplementary material for: Expression of Glypican 3 Is an Independent Prognostic Biomarker in Primary Gastro-Esophageal Adenocarcinoma and Corresponding Serum Exosomes
Source: J Clin Med. 2019 May 16;8(5):696. doi: 10.3390/jcm8050696 (PMC6572603; doi:10.3390/jcm8050696)
Supplement: Supplementary file 1 [file jcm-08-00696-s001.pdf]

## Supplementary Figure 1

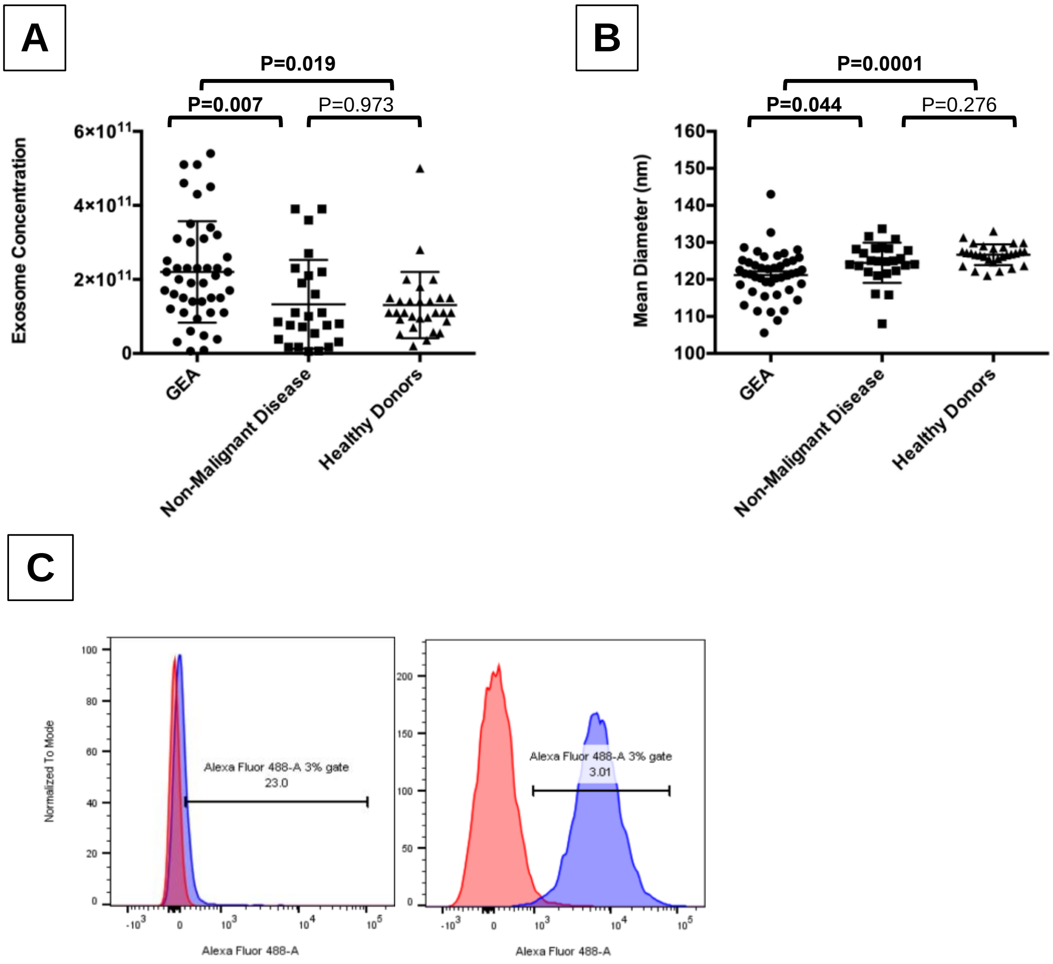


**Supplementary Figure 1. (A)** and **(B)** Dot plots showing the exosome concentration and mean exosome size of selected samples of 44 patients with GEA, 25 patients with non-malignant disease and 29 healthy donors. **(C)** Two representative histograms of GPC3 on exosome-bound latex beads isolated serum samples of a patient with histologically confirmed GEA (left panel) and a healthy donor (right panel) using flow cytometry.

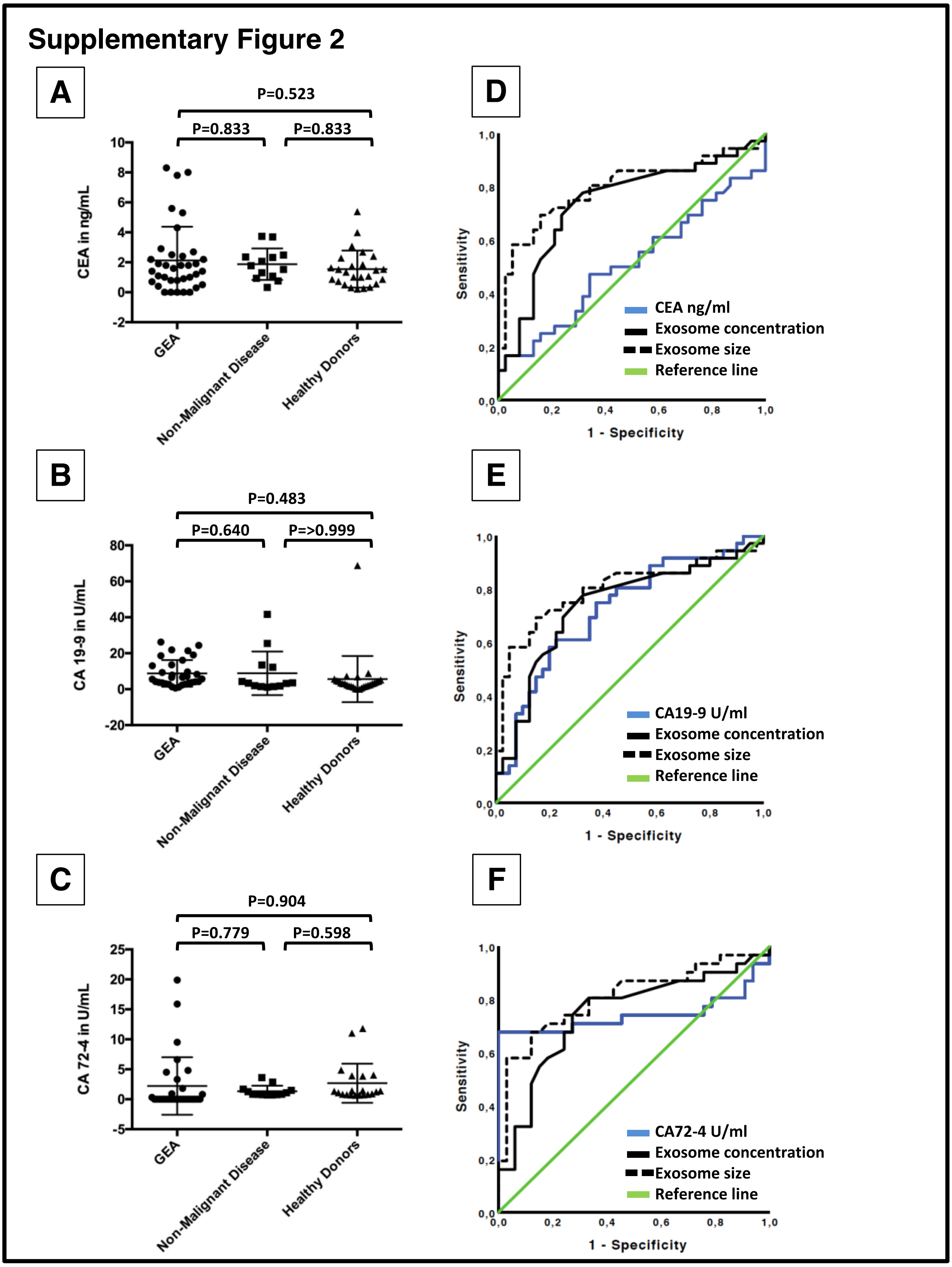


**Supplementary Figure 2. (A), (B) and (C)** Dot plots of the expression of CEA, CA 19-9, and CA 72-4 in human serum samples from patients with GEA, patients with non-malignant disease and healthy donors measured by immunoassays. **(D), (E) and (F)** ROC curves of the diagnostic sensitivity and specificity of the expression of CEA, CA 19-9 and CA 72-4 in human serum samples from patients with GEA vs. control subjects (non-malignant disease and healthy donors).

## Supplementary Figure 3

**A**

| Pairwise comparison of ROC curves | | | | | |
|---|---|---|---|---|---|
| Variable | CA19-9 | CA72-4 | CEA | eGPC3 | Combined Score |
| CA19-9 | N.A. | P = 0.65 | P = 0.03 | P = 0.02 | P = 0.004 |
| CA72-4 | | N.A. | P = 0.04 | P = 0.09 | P = 0.006 |
| CEA | | | N.A. | P < 0.0001 | P < 0.0001 |
| eGPC3 | | | | N.A. | P = 0.84 |
| Combined Score | | | | | N.A. |

**B** **Immunohistochemistry of GPC3**

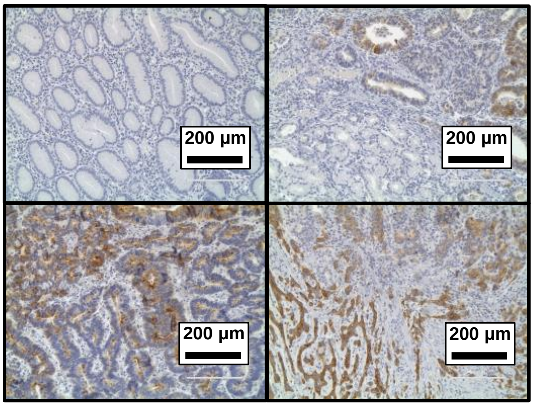


**C**

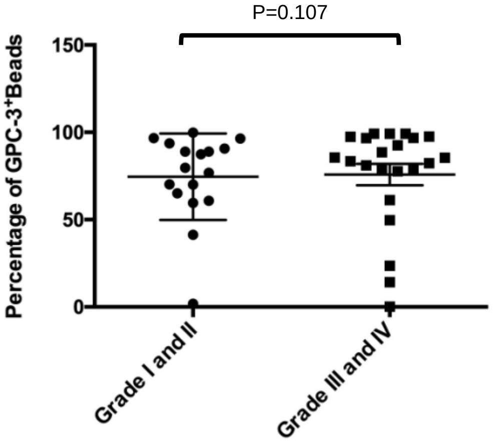


**D**

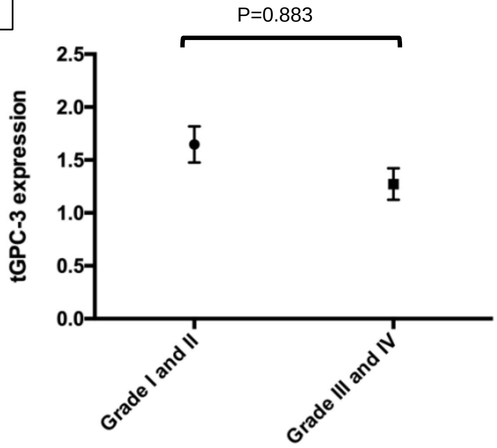


**E**

**Low-grade GEA**

| | tGPC-3 $^{low}$ | tGPC-3 $^{high}$ | P-Value |
|---|---|---|---|
| eGPC-3 $^{low}$ | 2 | 7 | 1.0 |
| eGPC-3 $^{high}$ | 2 | 6 | |

**F**

**High-grade GEA**

| | tGPC-3 $^{low}$ | tGPC-3 $^{high}$ | P-Value |
|---|---|---|---|
| eGPC-3 $^{low}$ | 4 | 6 | 0.19 |
| eGPC-3 $^{high}$ | 9 | 3 | |

**Supplementary Figure 3. (A) Pairwise** comparison of the AUC of ROC curves of selected markers including CA19-9, CA72-4, CEA, eGPC3 and a Combined Score. **(B)** Immunohistochemistry of GPC3 antibody on normal foveolar gastric mucosa (upper left panel) and GEA tissue. Representative images of GPC3 staining intensity of 1 (upper right panel), staining intensity of 2 (lower left panel) and staining intensity of 3 (lower right panel). **(C) and (D)** Dot plots of the association of the percentage of eGPC3+Beads (left panel) and tGPC3 expression (right panel) with tumor grade. **(E) and (F)** Association of eGPC3 and tGPC3 expression in relation to tumor grade in low-grade GEA (including grade I and II tumors, left panel) and high-grade GEA (including grade III and IV tumor, right panel).

## Supplementary Figure 4

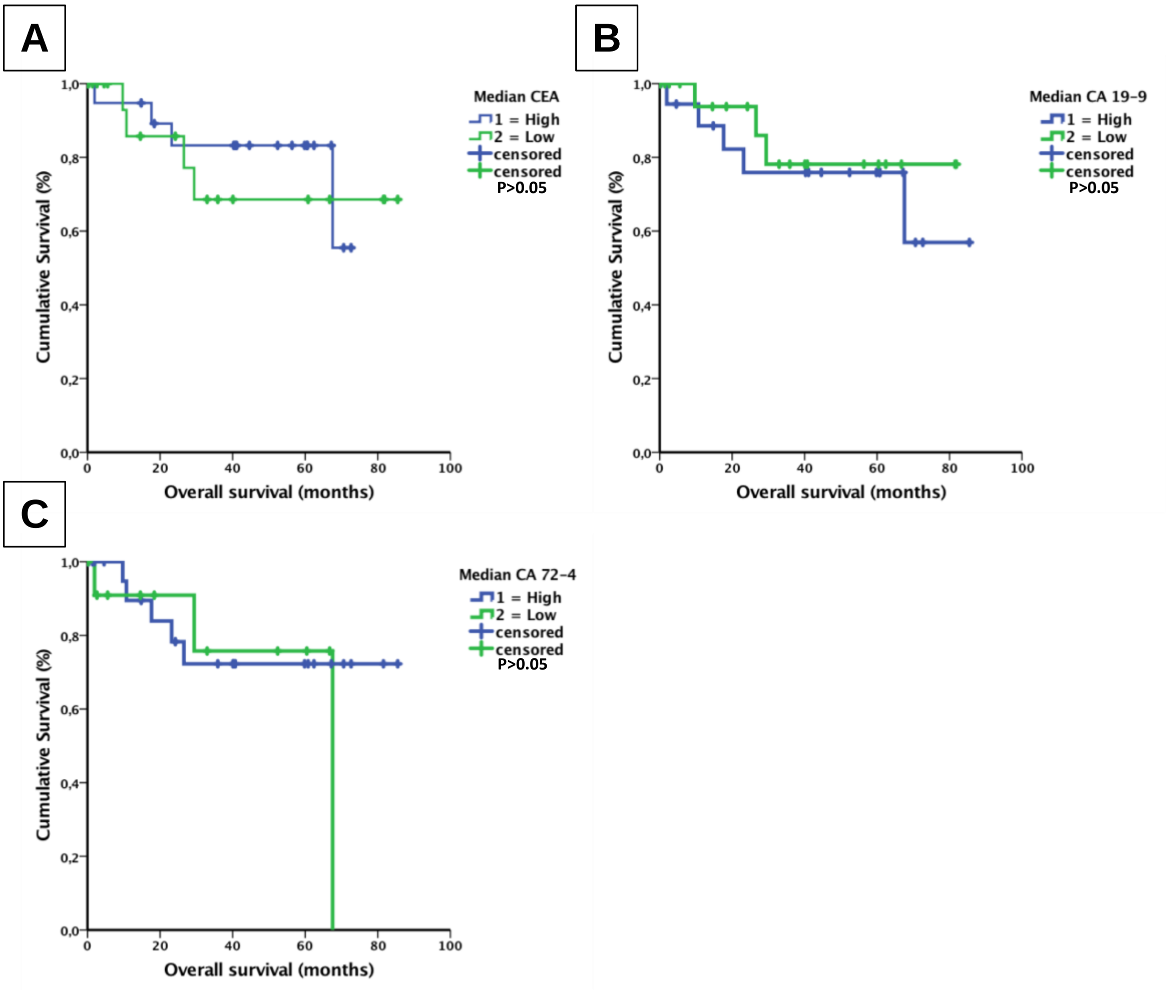


**Supplementary Figure 4.** Kaplan-Meier curves displaying overall survival in patients with GEA with high (blue line) and low (green line) expression of **(A)** CEA, **(B)** CA 19-9 and **(C)** CA 72-4 in blood serum samples. None of the three markers has been associated with significant dismal prognosis.
